# Hemostatic Cellulose Masquerading as a Postoperative Abscess Following Elective Colon Surgery: A Unique Case and Literature Review

**DOI:** 10.7759/cureus.90151

**Published:** 2025-08-15

**Authors:** Zamir A Shaikh, Caleb W Brown, Beth A Bulawa, Bracken Burns, Lou Smith

**Affiliations:** 1 Department of General Surgery, Alabama College of Osteopathic Medicine, Dothan, USA; 2 Department of General Surgery, East Tennessee State University’s Quillen College of Medicine, Johnson City, USA; 3 Department of General Surgery, Ballad Health Medical Associates, Greeneville, USA; 4 Department of Surgery, East Tennessee State University’s Quillen College of Medicine, Johnson City, USA; 5 Department of Surgery, University of Tennessee Medical Center, Knoxville, USA

**Keywords:** abdomino-pelvic ct, colon and rectal surgery, intra-abdominal abscess, postoperative complications, quality improvement (qi), surgicel™

## Abstract

Surgical site infections (SSIs) and intra-abdominal abscesses (IAAs) are common and costly complications following elective colon surgery, requiring prompt diagnosis and treatment. Hemostatic cellulose, a widely used intraoperative hemostatic agent, can mimic pathological lesions such as abscesses on imaging, leading to potential misdiagnoses. We present the case of a 68-year-old man who underwent elective colostomy reversal complicated by a postoperative computed tomography (CT) scan suggestive of an IAA. Upon review of the operative note and repeat imaging, the suspected abscess was determined to be a hemostatic cellulose. This case highlights the importance of meticulous intraoperative documentation and interdepartmental communication to prevent unnecessary interventions and improve patient outcomes. Additionally, it underscores the need for awareness among clinicians regarding this masquerading ability of hemostatic cellulose to avoid misinterpretation and its potential impact on both patients and hospital quality metrics.

## Introduction

Elective colon surgery continues to have the highest rate of surgical site infection (SSI) among all elective surgical procedures, with an infection rate of 13%-22%, while the overall SSI rate is only 0.5%-3% [1-3]. An estimated 70% of intra-abdominal abscesses (IAAs) are postsurgical, and as many as 6% of patients undergoing colorectal surgery will develop a postoperative abscess [4]. These infections span a continuum from superficial site infections (4%) to deep-seated intra-abdominal infections (12%) that can pose a serious threat to patient survival and are associated with significant morbidity, typically requiring at least a short duration of antibiotic therapy [5]. Larger abscesses often necessitate image-guided drainage or reoperative intervention, resulting in prolonged hospitalization and hospital readmission during the course of treatment. Consequently, SSIs and IAAs carry a substantial financial burden. The early recognition and accurate diagnosis of IAAs are important not only for optimal patient care but also for accurate quality metrics for individual surgeons and institutions.

Computed tomography (CT) is a valuable imaging tool commonly utilized in the diagnosis of IAA and often a component of image-guided drainage. However, CT can misidentify benign processes as pathological, such as an IAA [6]. We present a case in which hemostatic cellulose, a hemostatic agent requiring up to two months for reabsorption, was employed during elective colon surgery and later misidentified as IAA, and the resulting unique diagnostic and clinical management strategy was employed.

## Case presentation

**Figure 1:**
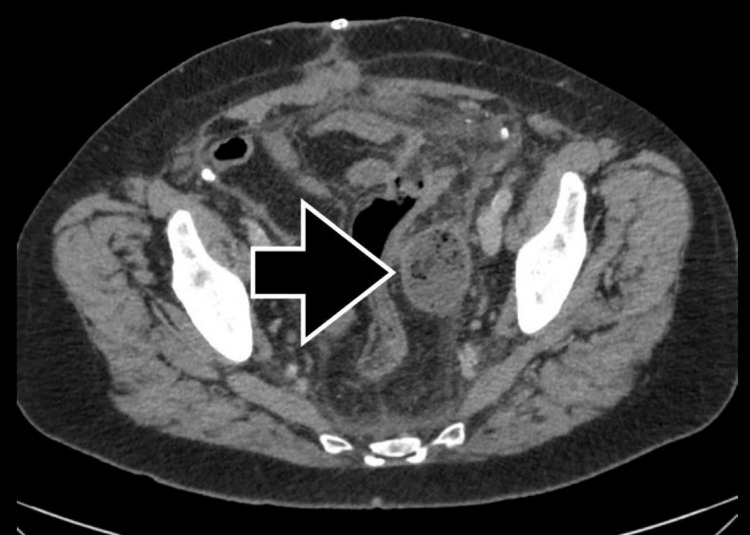
CT image from postoperative day 11 demonstrating an air-containing fluid collection measuring 3.6 × 4.6 × 4.8 cm in the left lateral pelvis. CT: computed tomography

A 68-year-old man presented for the elective reversal of a colostomy placed three months prior for acute diverticulitis complicated by perforation. The handsewn colorectal anastomosis was performed through an open abdominal incision, and a 5.1 × 10.2 cm piece of hemostatic cellulose was placed in the pelvis to control oozing. The patient was discharged on postoperative day 5 without any in-hospital complications. On postoperative day 6, the patient returned to the clinic complaining of fever, nausea, and loose stools. In the emergency department, the patient was hemodynamically normal and afebrile. Dry mucous membranes were noted on a physical examination. Laboratory testing revealed a WBC of 8.1 K/μL, a lactate of 1.4 mmol/L, and a procalcitonin of 0.09 ng/mL. Urinalysis was unremarkable, and blood cultures subsequently demonstrated no growth. A CT scan indicated a 3.6 × 4.6 × 4.8 cm encapsulated air-containing fluid collection in the left lateral pelvic sidewall consistent with an abscess (Figure 1).

**Figure 2:**
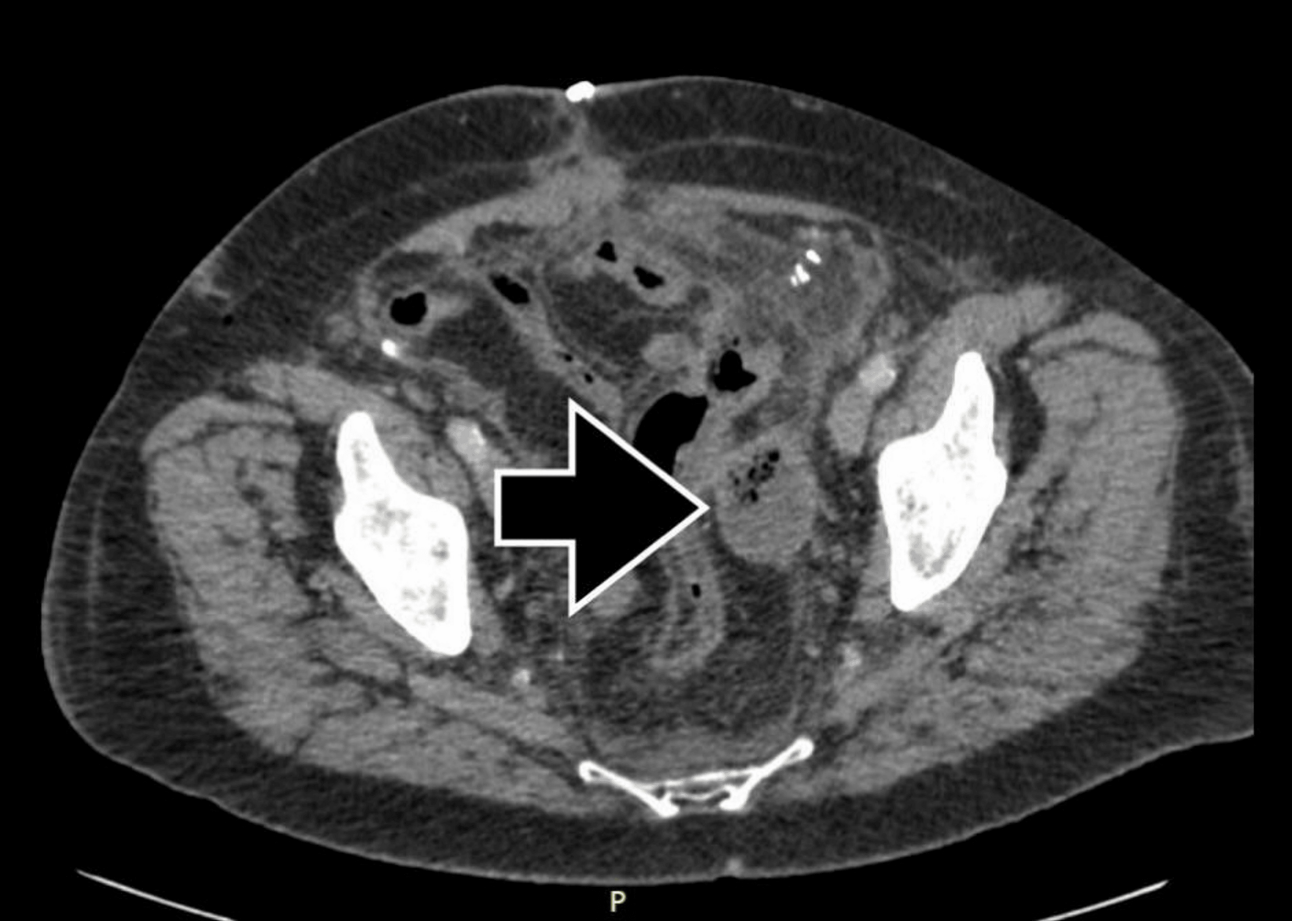
The air-containing fluid collection on postoperative day 14 measuring 3.4 × 4.4 × 4.8 cm.

Broad-spectrum antibiotics and intravenous (IV) fluids were administered with improvement in the patient’s clinical status. A repeat CT scan was performed three days after hospital readmission, and after discussing the patient’s clinical presentation with the surgeon and referencing the operative note, the radiologist reported a mild interval decrease in the size of the organized collection along the left pelvic sidewall, most likely representing Surgicel® packing (as referenced in the surgeon’s operative note). No definite abscess was identified (Figure 2).

**Figure 3:**
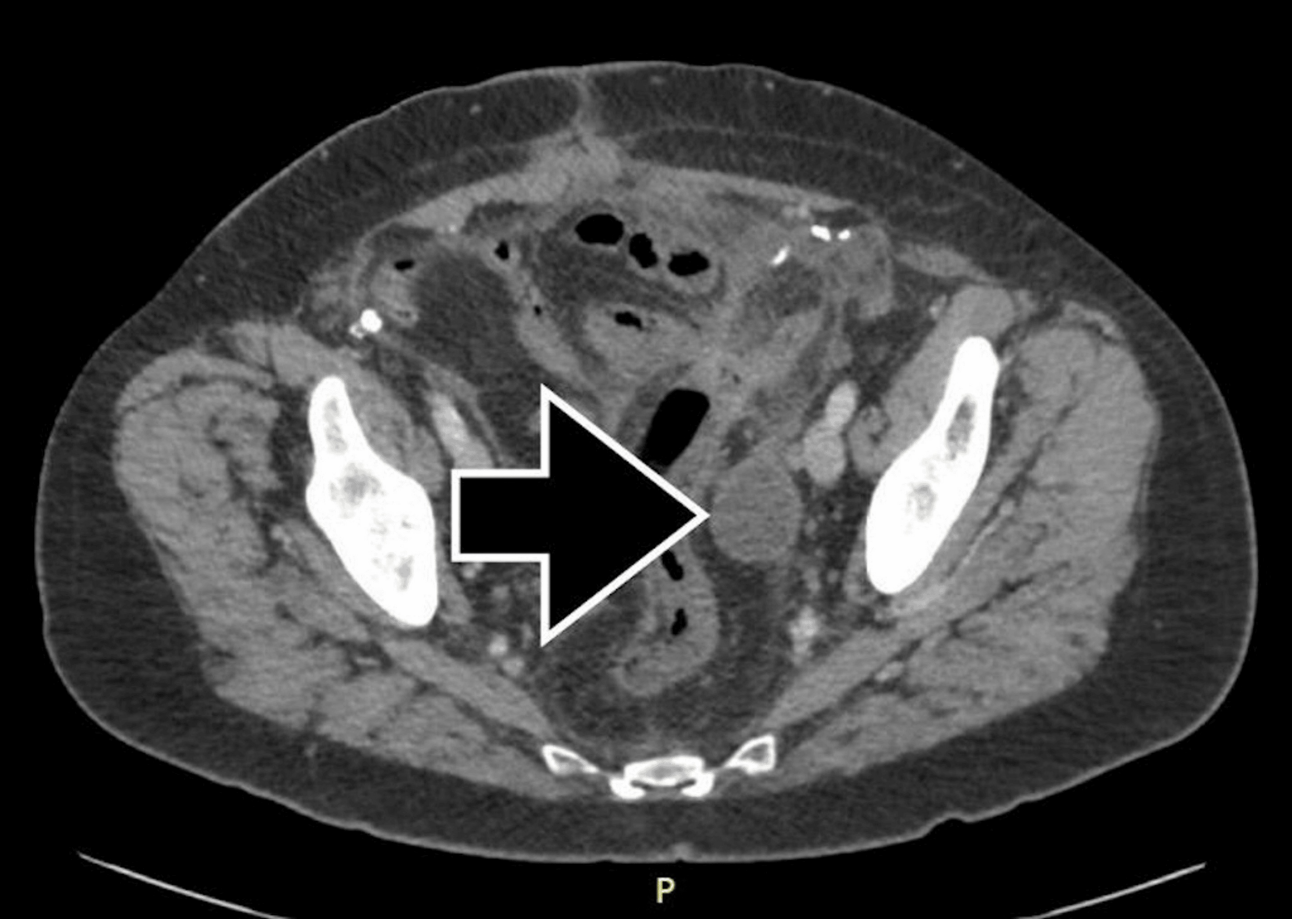
Persistence of the fluid collection on postoperative day 28 measuring 2.8 × 3.8.

In consultation with the operative surgeon, antibiotics were discontinued, and the patient was discharged in stable condition with the resolution of symptoms. Fourteen days following discharge, the patient returned to the hospital due to nausea and right upper quadrant abdominal pain and was diagnosed with acute cholecystitis. A third CT scan of the abdomen and pelvis was performed on postoperative day 28 prior to the surgical treatment of acute cholecystitis. Imaging incidentally revealed the persistence of the intra-abdominal fluid collection with a decrease in size to 2.8 × 3.8 cm (Figure 3). Following laparoscopic cholecystectomy, the patient was discharged and made a full recovery without further intervention.

## Discussion

Oxidized regenerated cellulose (ORC), commercially known as Surgicel®, is widely used across a number of different surgical disciplines as an intraoperative, topical hemostatic agent. The concept of topical hemostatic agents emerged in the early 20th century, with ORC entering the product market in 1960 [7]. ORC works to promote hemostasis physically by trapping and thus concentrating various blood components, such as red blood cells, platelets, and blood proteins, and chemically by lowering the pH to promote nonspecific platelet aggregation [8].

Although ORC is designed to be bioabsorbable, its radiographic appearance during degradation can mimic concerning postoperative complications such as abscesses, hematomas, lymphoceles, or even tumor recurrence [6,9]. In one blinded radiology study, ORC was correctly identified in only 11% of cases, with misinterpretations including abscess, hematoma, and lymphocele [6]. This diagnostic ambiguity has been documented in a variety of specialties.

In neurosurgery, for example, ORC has led to reoperation due to imaging features mimicking residual or recurrent tumor [9]. Similar cases have been reported in other surgical specialties as well. In Table 1, we summarize a collection of representative case reports and case series published over the course of 18 years that demonstrate the masquerading variability of ORC on postoperative presentation, not only in the pathology mimicked but also in the anatomical location, time to presentation, and management [9-17]. These findings are compared to the case described above in the final row.

**Table 1:** Reported cases of oxidized regenerated cellulose (ORC) mimicking postoperative pathologies across surgical specialties.

Authorship	Title	Publication Year	Presentation Time (Postoperative)	Pathology Mimicked	Management
Ribalta et al. [9]	Textiloma (gossypiboma) mimicking recurrent intracranial tumor	2004	1-7 months	Recurrent tumor (pituitary adenoma, tanycytic ependymoma, anaplastic astrocytoma, gliosarcoma, and oligodendroglioma)	Repeat operation
Somani et al. [10]	Surgicel granuloma mimicking a renal tumour	2006	Unspecified	Renal tumor	Not reported
Royds et al. [11]	Oxidized cellulose (Surgicel) based reaction post thyroidectomy mimicking an abscess: A case report	2012	30 days	Abscess	ORC removal
Wang and Chen [12]	Surgicel® (oxidized regenerated cellulose) granuloma mimicking local recurrent gastrointestinal stromal tumor: a case report	2013	4 months	Recurrent gastrointestinal stromal tumor	Exploratory laparotomy
Cormio et al. [13]	Surgicel^®^ granuloma mimicking ovarian cancer: A case report	2016	6 months	Ovarian cancer	Laparoscopic exploration
Kaneyuki et al. [14]	Surgicel® packing remnants mimicking mediastinitis after adult cardiac surgery	2018	13 days	Mediastinitis	Repeat operation
Regragui et al. [15]	Surgicel mimicking early onset prosthetic valve endocarditis: case report	2020	2 months	Endocarditis	Antibiotics
Adamicová et al. [16]	Gossypiboma mimicking recurrent mandibular tumor: case report	2020	2 months	Recurrent ameloblastoma	Clinical consultation and diagnosis correction
Turkyilmaz et al. [17]	Surgicel mimicking recurrent pelvic neuroblastoma in a 3-year-old boy	2022	6 months	Recurrent neuroblastoma	Exploratory laparotomy
Our case	Hemostatic Cellulose Masquerading as a Postoperative Abscess Following Elective Colon Surgery: A Unique Case and Literature Review	2025	11 days	Abscess	Conservative management with repeat imaging

While the masquerading abilities of ORC have been described in a number of different contexts, we believe that this is the first reported case of ORC mimicking an IAA following elective colon surgery, and it is this fact that makes this case of unique importance. The rate of postoperative infection and IAA formation is significantly greater following colon surgery as compared to other surgical procedures [1-4]. Because of the frequency and severity of these infections, there has been nearly a full century of efforts to define improved processes for prevention [18]. Numerous methods have been proposed and employed before and during elective colon surgery to prevent infection. Many have strong scientific foundations, while others are driven solely by expert opinion.

Currently, many surgeons and hospitals employ the recommendations of enhanced recovery after surgery (ERAS) as a perioperative pathway to reduce hospital length of stay (LOS) and postoperative complications, including infections such as SSI and IAA formation. The American College of Surgeons has developed the National Surgical Quality Improvement Program (NSQIP), a database that follows variables from participating hospitals in order to enhance outcomes from high-risk surgeries, such as those of the colon. NSQIP has shown that a colon protocol reduces mortality, infection rates, septic shock, and unplanned intubations [19]. In addition to national efforts to reduce infections from colon surgery, hospitals employ departments of infection control to follow metrics and make improvements in overall care. Their role in improvement involves interfacing with individual surgeons and reporting overall hospital results in online formats visible to potential and current patients.

Considering the strong emphasis placed on preventing postoperative infections following colon surgery as described, it is not difficult to see why the index of clinical suspicion for the presence of a postoperative abscess in the above patient would be remarkably high. However, this case, in the context of the others described above, suggests that radiology suggesting an IAA in a high-risk situation does not guarantee the diagnosis. Furthermore, while the consequences of the misdiagnosis of benign radiologic findings as pathology may seem less significant in certain cases (e.g., unnecessary administration of antibiotics), in other cases, the intervention places the patient at risk of significant harm while providing no reciprocal benefit (e.g., invasive procedure or reoperation). This concern is underscored by a study suggesting a reoperation rate as high as 41.2% in patients with postoperative intra-abdominal septic complications such as IAA within one year of their index operation [20].

The patient described in this case report illustrates this point. His surgical operation was performed, and his follow-up care was provided at a rural hospital with limited specialized care, such as interventional radiology. While empiric antibiotics were clinically responsible in a high-risk situation, if the patient’s clinical presentation had not improved with conservative management (e.g., IV fluids) and the escalation of care had been deemed necessary, reoperation would have been the likely outcome. This outcome would have placed the patient at risk of a surgical or anesthesia complication while providing no benefit. Furthermore, a misdiagnosis and the potential negative outcome would have detrimentally affected both surgeon and institutional quality of care metrics, considering the strong emphasis placed on postoperative infectious complications.

In light of this case and the above discussion, we recommend that surgeons not only remain mindful of the masquerading ability of ORC but also take the specific intentional steps to mitigate, where possible, a radiologic misdiagnosis. First, use the minimal effective amount of ORC, and remove excess material when feasible. Second, clearly document the use and exact location of hemostatic agents in operative reports. Third, include ORC in the radiologic differential diagnosis for early postoperative collections. Finally, encourage prompt, collaborative review between surgical and radiology teams when discrepancies arise at the earliest opportunity. This case contributes to the growing body of literature on ORC-related imaging confusion and underscores the need for clinical-radiologic correlation to not only optimize patient care but also avoid detriment to surgeon and institutional quality of care metrics.

## Conclusions

This case highlights the potential for oxidized regenerated cellulose (ORC) to mimic serious postoperative complications such as IAA, particularly in high-risk surgeries such as elective colon procedures. Despite characteristic imaging findings, the patient’s stable clinical status and careful review of operative documentation allowed for the correct identification of retained ORC, thus avoiding any additional unnecessary interventions. Given the significant morbidity, cost, and impact on institutional quality metrics associated with misdiagnosis, clinicians must maintain a high index of suspicion for retained hemostatic agents in the differential diagnosis of postoperative collections. Clear documentation, interdepartmental communication, and clinical-radiologic correlation are critical in ensuring accurate diagnosis and optimizing patient outcomes.
